# The mechanisms and prediction of non-structural carbohydrates accretion and depletion after mechanical wounding in slash pine (*Pinus elliottii*) using near-infrared reflectance spectroscopy

**DOI:** 10.1186/s13007-022-00939-2

**Published:** 2022-09-01

**Authors:** Yanjie Li, Honggang Sun, Thiago de Paula Protásio, Paulo Ricardo Gherardi Hein, Baoguo Du

**Affiliations:** 1 Research Institute of Subtropical Forestry, Chinese Academy of Forestry, Hangzhou, 311400 China; 2 Federal Rural University of Amazonia-UFRA, Campus Parauapebas, Parauapebas, Pará 68515-000 Brazil; 3 Department of Forest Science, Federal University of Lavras-UFLA, Lavras, Minas Gerais 37200-900 Brazil; 4 Chair of Tree Physiology, Institute of Forest Sciences, AlbertLudwigs-Universitat Freiburg, Georges-Koehler-Allee 53, 79110 Freiburg, Germany

**Keywords:** Non-structural carbohydrate, Vibrational spectroscopy, Mechanical wounding, Slash pine

## Abstract

**Background:**

The allocation of non-structural carbohydrates (NSCs) plays a critical role in the physiology and metabolism of tree growth and survival defense. However, little is known about the allocation of NSC after continuous mechanical wounding of pine by resin tapping during tree growth.

**Results:**

Here, we examine the NSC allocation in plant tissues after 3 year lasting resin tapping, and also investigate the use of near-infrared reflectance (NIR) spectroscopy to quantify the NSC, starch and free sugar (e.g., sucrose, glucose, and fructose) concentrations in different plant tissues of slash pine. Spectral measurements on pine needle, branch, trunk phloem, and root were obtained before starch and free sugar concentrations were measured in the laboratory. The variation of NSC, starch and free sugars in different plant tissues after resin tapping was analyzed. Partial least squares regression was applied to calibrate prediction models, models were simulated 100 times for model performance and error estimation. More NSC, starch and free sugars were stored in winter than summer both in tapped and control trees. The position of resin tapping significantly influenced the NSCs allocation in plant tissues: more NSCs were transformed into free sugars for defensive resin synthesis close to the tapping wound rather than induced distal systemic responses. Models for predicting NSC and free sugars of plant tissues showed promising results for the whole tree for fructose (R^2^_CV_ = 0.72), glucose (R^2^_CV_ = 0.67), NSCs (R^2^_CV_ = 0.66) and starch (R^2^_CV_ = 0.58) estimates based on NIR models. Models for individual plant tissues also showed reasonable predictive ability: the best model for NSCs and starch prediction was found in root. The significance multivariate correlation algorithm for variable selection significantly reduced the number of variables. Important variables were identified, including features at 1021–1290 nm, 1480, 1748, 1941, 2020, 2123 and 2355 nm, which are highly related to NSC, starch, fructose, glucose and sucrose.

**Conclusions:**

NIR spectroscopy provided a rapid and cost-effective method to monitor NSC, starch and free sugar concentrations after continuous resin tapping. It can be used for studying the trade-off between growth and production of defensive metabolites.

## Background

Asynchronization of carbon gain through photosynthesis with carbon demand for growth, respiration, reproduction and defense determines the strategy for allocation of non-structural carbohydrates (NSCs) in woody plants [1, 2]. NSC reserves are versatile resources for metabolic processes, particularly when mobile carbon gain is limited under unfavorable environmental conditions [3]. Stored carbohydrates in all plant species comprise structural carbohydrates (SCs) and NSCs. SCs include celluloses and hemicelluloses(holocellulose) which are essential components for plant morphological construction, and these compounds are not available to the plant for future use because plants lack enzymes for cellulose degradation [4]. NSCs are accumulated and stored as starch and free sugars (e.g., glucose, sucrose and fructose) that support primary metabolism and growth [1, 5, 6]. NSCs can undergo frequent transformations, as the current assimilation is inadequate to meet the carbon requirement for physiological maintenance and growth under the current scenario of season variation [7]. In addition to these roles, NSCs are also the original sources of secondary metabolites with defensive functions [8]. Under external stress or stimulus in a short time, like insect attack, tree defensive systems are activated and secondary defensive chemicals are produced through specialized biosynthetic pathways; correspondingly, NSCs that might otherwise have been allocated to growth and reproduction will be diverted to produce defensive exudates [9].

Slash pine is an important species originating from the North America and introduced to southern Europe, southeast Asia, and south America [10, 11]. Owing to its remarkable characteristics, i.e., rapid growth, wide adaptability, and high resin yield [12], it is thus a major timber and resin tapping species in China which the current plantation area and oleoresin exceeds 1.2 million ha and 1.3 × 10^6^ metric ton in 2018 [13]. Pine resin mainly consisted of monoterpene rich turpentine and diterpenoid resin acids or rosin has a wide range of industrial uses [14]. However, resin tapping year after year may aggravate the risk of broken stems at the resin tapping position [15], inhibit growth processes [16, 17], decrease germination success [18] and increase susceptibility to attack by insects and their fungal associates [19]. Better understanding of NSC allocation patterns, particularly seasonal patterns after resin tapping can provide valuable information on defense, trade-off between growth and survival, and give advice for forest management [18, 20].

This disagreement might be attributed to the methods of measuring NSC concentrations in plant tissues [6], which are time-consuming and expensive and limit the scope for NSC research on large numbers of samples. Near infrared reflectance (NIR) spectroscopy is a rapid, cost-effective and high-throughput technique that can be used for measuring organic compounds in plant tissues [21, 22]. For instance, NIR based models yielded promising predictions for N, neutral detergent fiber, lignin and cellulose concentrations in leaves of 17 different woody species [23]. The starch concentrations in *Sorghum bicolor* (sorghum grain) and *Rumex obtusifolius* roots have been also successfully predicted by NIR [24, 25]. Encouraging results were reported for predictive models to estimate the NSC in *Toona ciliata* wood from NIR spectra directly recorded on wood discs and milled wood samples [22]. Furthermore, spectroscopic determination of NSC concentration in different tissue types with seasonal change of different tree species was also achieved [26]. Last years is fast developing methods of non-destructive measurements regarding presence of specific secondary metabolites and well developed to study correlation between results from hyper-spectral imaging analysis, fluoresence imaging or near-infrared spectroscopy analysis and wet biochemical estimation [27–29]. However, little is known on pine trees. Therefore, we hypothesized that (1) the NSC concentrations in pine tissues can be successfully predicted by NIR technology; (2) the concentration of NSCs in tree leaves, branches, trunks and roots would respond differentially to resin tapping in different seasons; and (3) the dynamic responses of NSC concentrations in plant tissues would depend on the position of resin tapping, with stronger responses near the resin tapping area.

## Materials and methods

### Study sites and experimental design

This study was conducted in the Tianmu Mountains in Hangzhou, China (30° 42′ N, 120° 30′ E). This region has a subtropical monsoon climate with long warm summers and short cool winters. Mean annual, mean monthly minimum (January) and mean monthly maximum (July) temperatures are 16.7 °C, 5.6 °C and 27.3 °C, respectively. The average length of the growing season is 246 frost-free days, while precipitation, including rain and snow, averages 1697 mm.

The experimental plantation was established in January 1993 at three sites (site 1: 20.7 m altitude, eastern slope, 25% gradient; site 2: 22.0 m altitude, southeast slope, 23% gradient; site 3: 23.1 m altitude, southwestern slope, 20% gradient). Each site was planted with 2.0 m between rows and 2.0 m between trees within a row. Each row contained 6 seedlings and there were 30 rows in each site. A 3-row buffer zone consisting of similarly planted seedlings surrounded each site.

The number of surviving stems and growth vigor within each row were observed towards the end of 2013. Both control trees (without tapping) and tapped trees were selected randomly in plots site 1, site 2 and site 3, and the number of control trees and tapped trees were 12/42, 10/23 and 11/23, respectively. In total, 121 trees were selected.

### Sample collection

Tapping was performed from mid-May to mid-October (mean monthly air temperature above 10 °C) in 2014 and 2016 according to the bark chipping method [31, 30]. The downward bilateral tapping method was applied to obtain crude resin [32]. Resin tapping was conducted in the uphill tree face at 2.0 m above the ground and using about 40% of the trunk circumference. Tapping was conducted every two days and the length of the tapping face was limited to 20–25 cm along the stem in each year. All the tapped trees and control trees without tapping were sampled for carbohydrate analysis in the summer (mid-July) and winter (mid-December) 2017. At each sampling time, root, trunk phloem, branches and needles were collected. Root samples distance from 10 cm soil depth were fully consisted of fine roots with diameter < 2 mm. For bole samples, two 8 cm × 3 cm stem phloem strips were extracted using chisel and mallet above the tapping face and exactly the same on the opposite side. Newly needle, newly branch, one-year-old needle and one-year-old branch without visible insect/pathogen injury were sampled from the middle crowns using a 20-m-high retractable pruning shears. All samples were stored on dry ice immediately after collection and sent to the laboratory to avoid tissue respiration. Samples were dried at 60 °C for 72 h to obtain constant weight and then ground into powder using a Wiley Mini Mill (Thomas Scientific, Swedesboro, NJ, USA) with a 0.5-mm aperture mesh sieve.

### NIR spectral records

NIR spectra of each sample were recorded in reflectance mode using a NIR spectrometer (Analytical Spectral Devices, Boulder, CO, USA) equipped with a NIR fiber-optic probe. NIR signature were recorded from 1100 to 2500 nm with a spectral resolution of 8 nm (totaling 175 wavelength), and 32 scans were averaged per spectrum. Three spectral measurements were collected for all plant samples and were averaged to determine the mean spectrum per sample. Each mean spectrum was then converted to an absorbance spectrum (log 1/R, where R = reflectance) for model use. In total, 1985 samples were used. Standard normal variate (SNV) + first derivative transformation using Savitzky–Golay smoothing (13-point filter) was applied on the NIR spectra in order to reduce noise and to calibrate and cross-validate NIR models.

### Analysis of concentrations in plant tissue samples

The details of NSC content measurement was described by Hoch, et al. [33]. About 20 mg (0.1 mg precision) of dried plant tissue powder and 2 mL deionized water were boiled together for 30 min, and then centrifugated for 5 min with 8000 rpm, after that, the supernatant was extracted 500 μL to a new micro tube and treated with phosphoglucose-isomerase and invertase (Sinopharm Chemical Reagent Co., Ltd, Beijing, China) to convert fructose and sucrose into glucose. A microplate photometer (BioTek Epoch; BioTek Instruments, Inc., Winooski, VT, USA) was used to measure the total glucose concentration at 340 nm after conversion of glucose to glucose 6-phosphate with the glucose hexokinase assay (G3 292, Sigma), and the rest of water was incubated to a crude fungal amylase (Sinopharm Chemical Reagent Co., Ltd, Beijing, China) at 40 °C for 15 h to decompose starch to glucose, finally, the total glucose concentrations were determined.

The difference between the concentration of free sugars (i.e., glucose, fructose and sucrose) and the measured total glucose concentration after digestion of starch were calculated as the starch concentration. In order to ensure reliablity of the measured data in the laboratory, the *Citrus* leaves (Institute of Geophysical and Geochemical Exploration of China, Beijing, China) were analyzed to check the replicability of glucose determination and the Pure glucose, fructose, and sucrose solutions were used for calibrations. NSC concentrations were measured as a percentage of dry matter.

### NIR modeling of free sugars, starch and total NSC in different plant tissues

Differences among plant tissues might influence the effectiveness of NIR spectra to calibrate prediction models for NSC, free sugars and starch. Therefore, samples were divided into four groups of plant tissues, namely trunk (968 samples), root (286 samples), needle (484 samples) and whole tree mixed plant tissues. The two sides of stem phloem from the trunk, newly branch and one-year-old branch were summed to give the trunk group, and the newly needle and one-year-old needle were considered as the needle group.

NIR spectra and the measured values of free sugars, starch and NSC in plant tissues were used to develop the predictive models. In each group, samples were separated into a calibration set (80%) and validation set (20%) by random sampling and testing the model with 100 simulations to evaluate the model performance, including the overall model stability and the uncertainty of predictions [31]. Partial least squares regression (PLSR), using leave-one-out cross-validation and a variable selection algorithm called the significance multivariate correlation (sMC) filter method, was used to calibrate models and to find the most important spectral variables [34, 35]. The coefficient of determination (R^2^) and the root-mean-squared error (RMSE) were used to establish the best sMC-PLSR model for end use. Statistical analyses were performed in R software (version 3.1.2) [36] using the following packages: pls [37] for PLSR model calibration, prospectr [38] for the pre-processing method, plsVarSel [34] for selection of sMC variables, ggplot2 [39] for graph plots and asreml-R package [40] for statistical analyses.

**Fig. 1:**
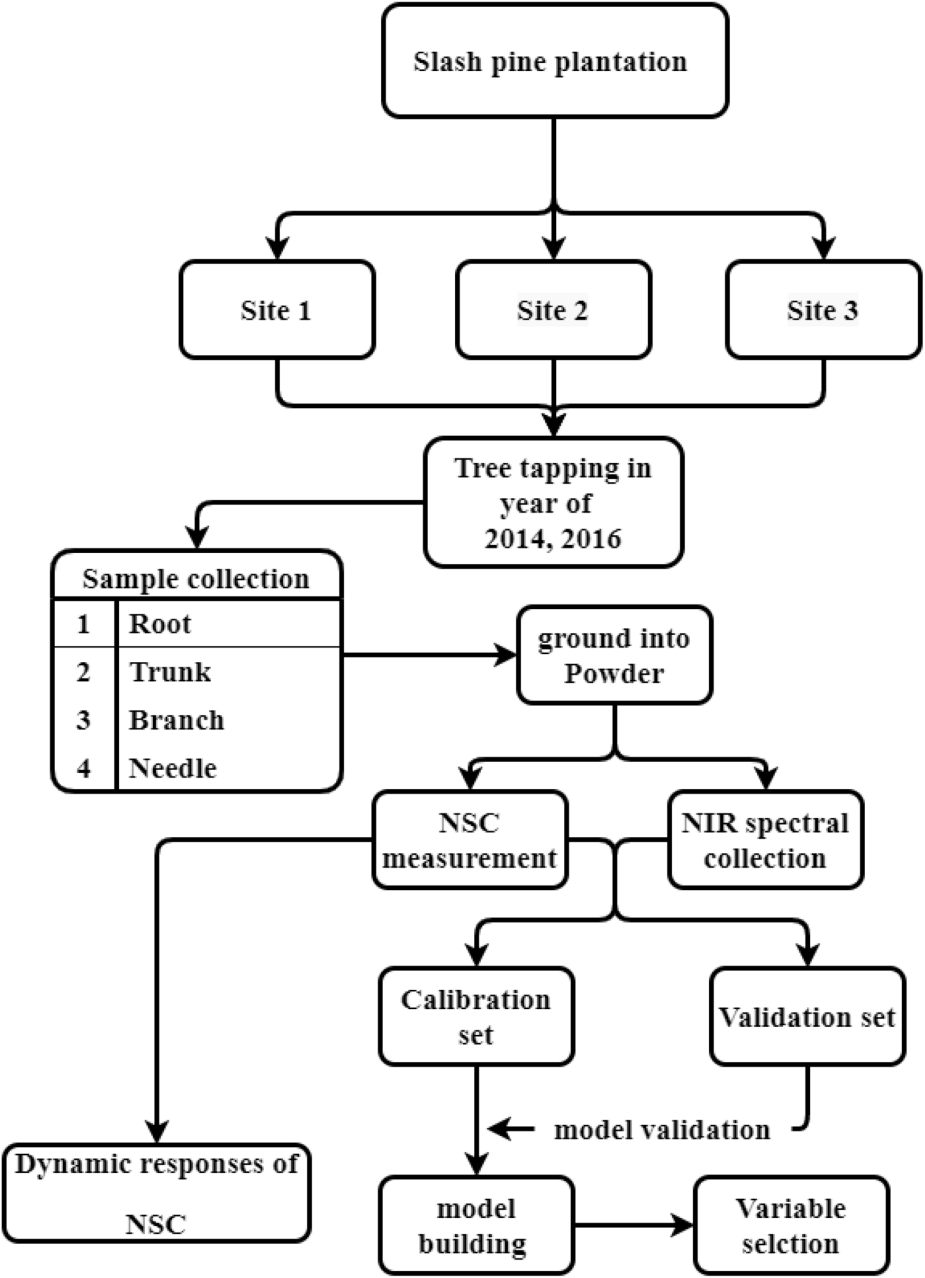
The workflow to collect the data from tree samples and used for NSC modelling using the NIR spectral

The framework follow chart were plotted in Fig. 1.

## Results

### NSC distribution after resin tapping

**Fig. 2:**
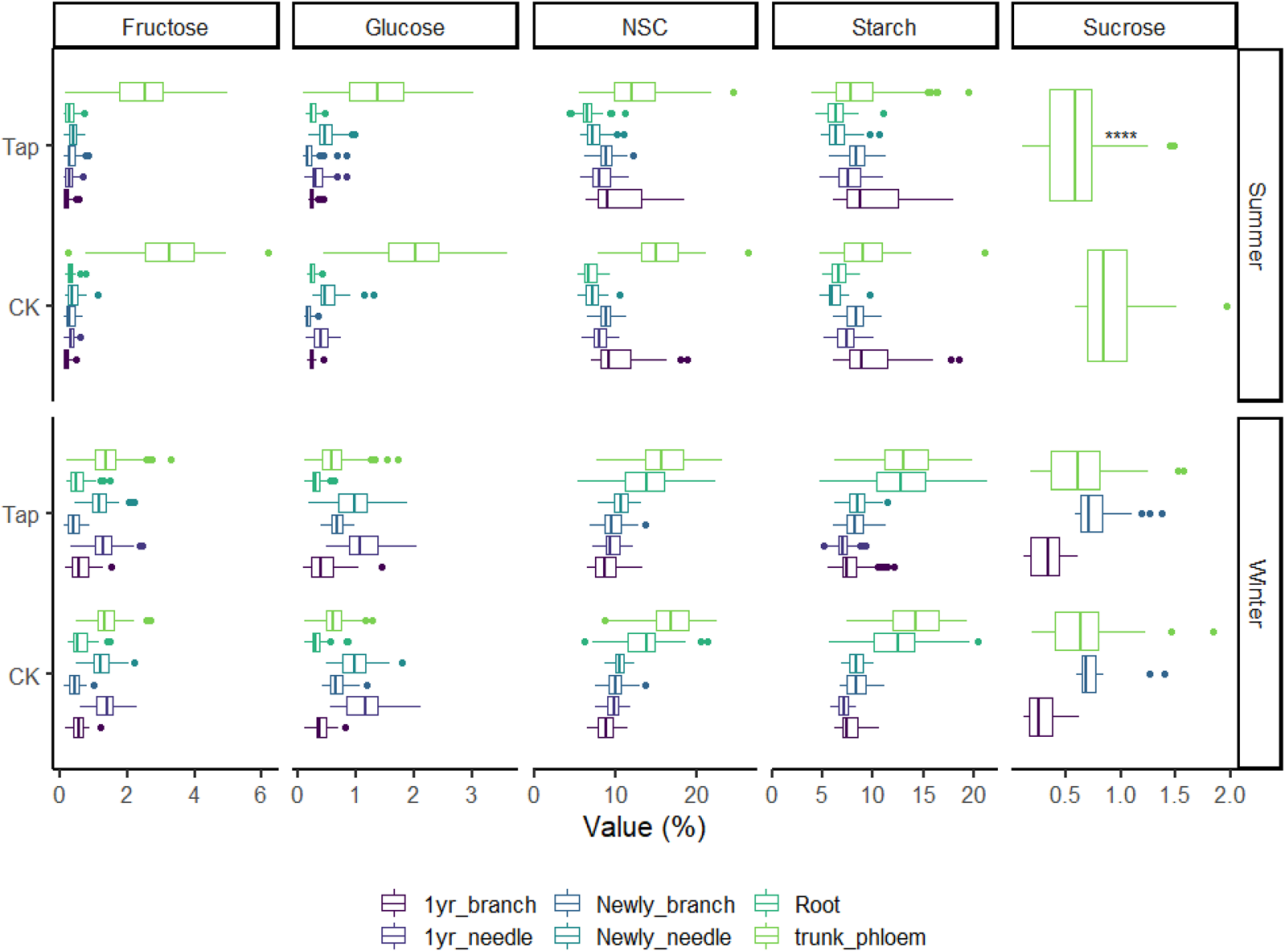
Distribution of NSC, starch, glucose, fructose and sucrose concentrations in different plant tissues from tapped and control trees in summer and winter. *CK* control tree, value (%): concentrations (based in the dry mass), Tap: tapped tree, black spots: outlier samples. *Represent the significant between CK and TAP, *p <  = 0.05, *p <  = 0.01, ***p <  = 0.001

**Fig. 3:**
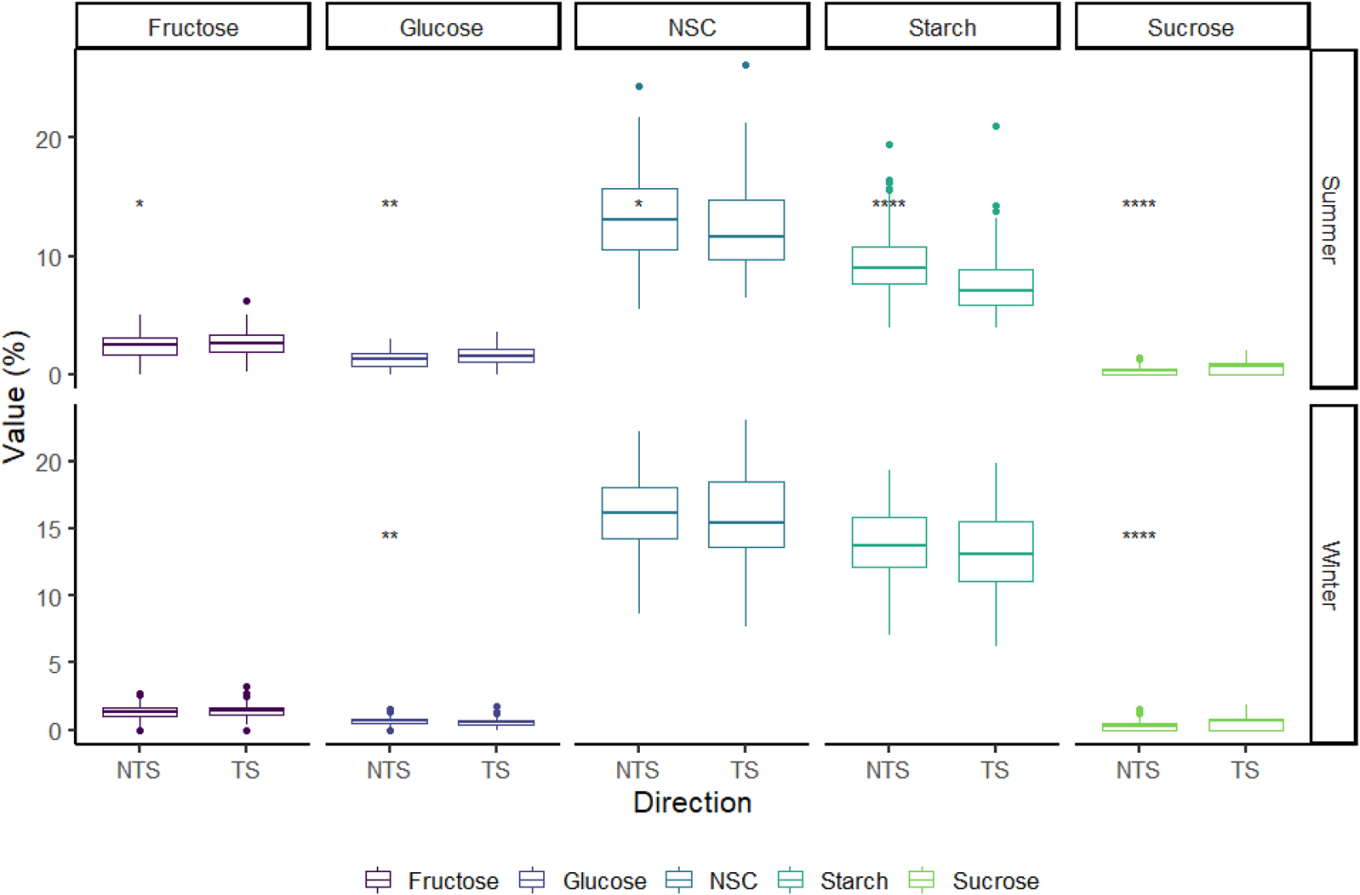
Distribution of NSC, starch, glucose, fructose and sucrose in trunk phloem from tapping trees in summer and winter. *NTS* non-tapping side, *TS* tapping side, value (%): concentrations (based in the dry mass), black spots: outlier samples. *Represent the significant between NTS and TS, *p <  = 0.05, *p <  = 0.01, ***p <  = 0.001

In summer, the trunk phloem and one-year-old branch had the highest starch concentration (mean of 11.05% and 9.03%) and total NSC concentration (mean of 14.40% and 9.68%) among the various plant tissues. Resin tapping trees showed lower starch concentration and NSC concentration than control trees in the trunk phloem (mean of 10.7%, 14% vs mean of 12.6%, 16.2%), but similar in root (mean of 9.64%, 10.2% vs mean of 9.51%, 10.1%), one-year-old branch (mean of 9.03%, 9.70% vs mean of 8.97%, 9.63%). In winter, the starch concentration and total NSC concentration were highest in the trunk phloem (mean of 13.50% and 15.91%) and root (mean of 12.73% and 13.56%); the trunk stored less in resin tapping trees than in control trees but the root showed the opposite patterns. The sucrose concentration did not show a large variation between tapping and control trees in summer or winter (Fig. 2). Trees stored the highest NSC concentration (mean of 14.4%) in the trunk phloem, containing the highest amounts of starch (mean of 11.1%), fructose (mean of 1.93%), glucose (mean of 1.03%) and sucrose (mean of 0.63%). In tapping trees, the tapping side stored less NSC (mean of 14.1%) and starch (mean of 10.5%) than the non-tapping (mean of 14.7% and 11.6%) side in both summer and winter; fructose and glucose were slightly higher on the tapping side than the non-tapping side in summer, but similar in winter (Fig. 3). All the different do not significantly between season and type of NSCs.

### Correlation among NSC, starch and free sugars in plant tissues

Glucose, sucrose and fructose were highly positively correlated with each other in the trunk and newly branch. Strong positive correlation was found between NSC and starch and between glucose and sucrose in every plant tissue respectively (Table 1). There was a significantly negative correlation between starch, glucose and fructose in trunk phloem and 1-year old branch but not significant in other plant tissues. NSC presents a highly positive correlation with glucose, fructose and sucrose in newly branch and with glucose and fructose in needles but not found significance in trunk phloem and root (Table 1).

**Table 1:** Correlation matrix describing relationships among NSC, starch and free sugars in plant tissues of resin tapping slash pine trees

		Glucose	Sucrose	Fructose	Starch
Trunk phloem	Sucrose	**0.13**			
	Fructose	**0.96**	**0.14**		
	Starch	**− 0.48**	0.01	**− 0.48**	
	NSC	**− **0.06	0.08	− 0.06	**0.9**
Newly branch	Sucrose	**0.42**			
	Fructose	**0.85**	**0.34**		
	Starch	**0.23**	0.23	0.11	
	NSC	**0.45**	**0.31**	**0.34**	**0.97**
Newly needle	Sucrose	–			
	Fructose	**0.91**	–		
	Starch	**0.42**	–	**0.52**	
	NSC	**0.71**	–	**0.79**	**0.93**
1 yr branch	Sucrose	− 0.06			
	Fructose	**0.9**	− 0.13		
	Starch	**− 0.18**	− 0.03	**− 0.29**	
	NSC	0.01	− 0.07	− 0.1	**0.98**
1 yr needle	Sucrose	–			
	Fructose	**0.95**	–		
	Starch	− 0.12	–	**− 0.13**	
	NSC	**0.64**	–	**0.64**	**0.67**
Root	Sucrose	–			
	Fructose	**0.85**	–		
	Starch	0.03	–	0.01	
	NSC	0.12	–	0.1	**0.98**

Bolded values are statistically significant at P < 0.05

### Prediction of NSCs by mixed tissues model

**Fig. 4:**
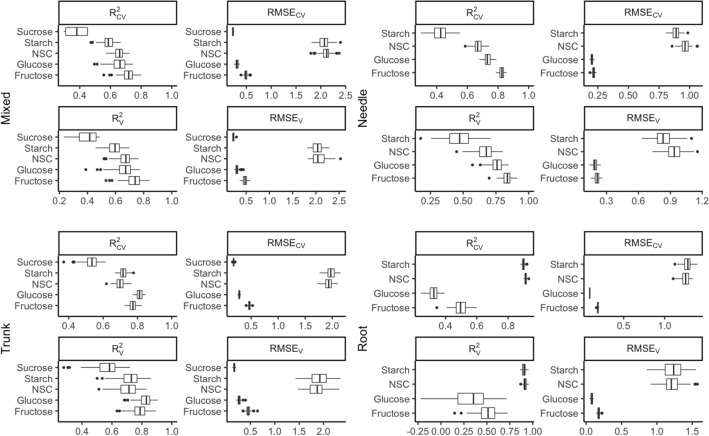
Distribution (95% confidence intervals) of calibration and validation statistics from 100 simulations for models predicting NSC, starch, glucose, fructose and sucrose in plant tissues. Each model permutation included 80% of the data for calibration and the remaining 20% for validation. R^2^_CV_: coefficient of determination of cross-validation; R^2^_v_: coefficient of determination of prediction when the model was applied to the validation data set; RMSE_CV_: root-mean-square error of cross-validation; RMSE_v_: root mean-square error of prediction when the model was applied to the validation data set

The mixed tissues prediction model was found to be the highest-performing for predicting fructose concentration, with a mean R^2^_CV_ of 0.72 and RMSE_CV_ of 0.49%, followed by predictions for glucose, NSC, starch and sucrose (Fig. 4). NSC was moderately accurately predicted by the mixed tissues model, with a mean R^2^_CV_ of 0.66 and RMSE_CV_ of 2.12% and RMSE_V_ of 2.05% for validation. When predicting starch, the mixed tissues model produced a mean R^2^_CV_ of 0.58, RMSE_CV_ of 2.09% and RMSE_V_ of 2.05% for external validation. The mixed tissues model showed the worst accuracy for sucrose prediction, with a mean R^2^_CV_ of 0.38 and RMSE_CV_ of 0.11%.

### Prediction of NSCs by separate tissue models

Data from three types of plant tissue, namely trunk, root and needle, were separated and considered as single-tissue models for NSC prediction. The best model for NSC and starch prediction was found in the root, with a mean R^2^_CV_ of 0.91 and 0.90, RMSE_CV_ of 1.25% and 1.28%, and RMSE_V_ of 1.23% and 1.22%, respectively, followed by the trunk and needle models. Sucrose prediction was not considered in the root and needle models because only trace amounts of sucrose were found. Starch showed a poor prediction in the needle model, with mean R^2^_CV_ and R^2^_V_ of only 0.41 and 0.48 respectively. The trunk and needle models showed satisfactory results for glucose and fructose predictions, with R^2^_CV_ of 0.83 and 0.79, respectively, in the trunk and 0.73 and 0.83 in the needle (Fig. 4).

### Variable selection and model optimization

**Fig. 5:**
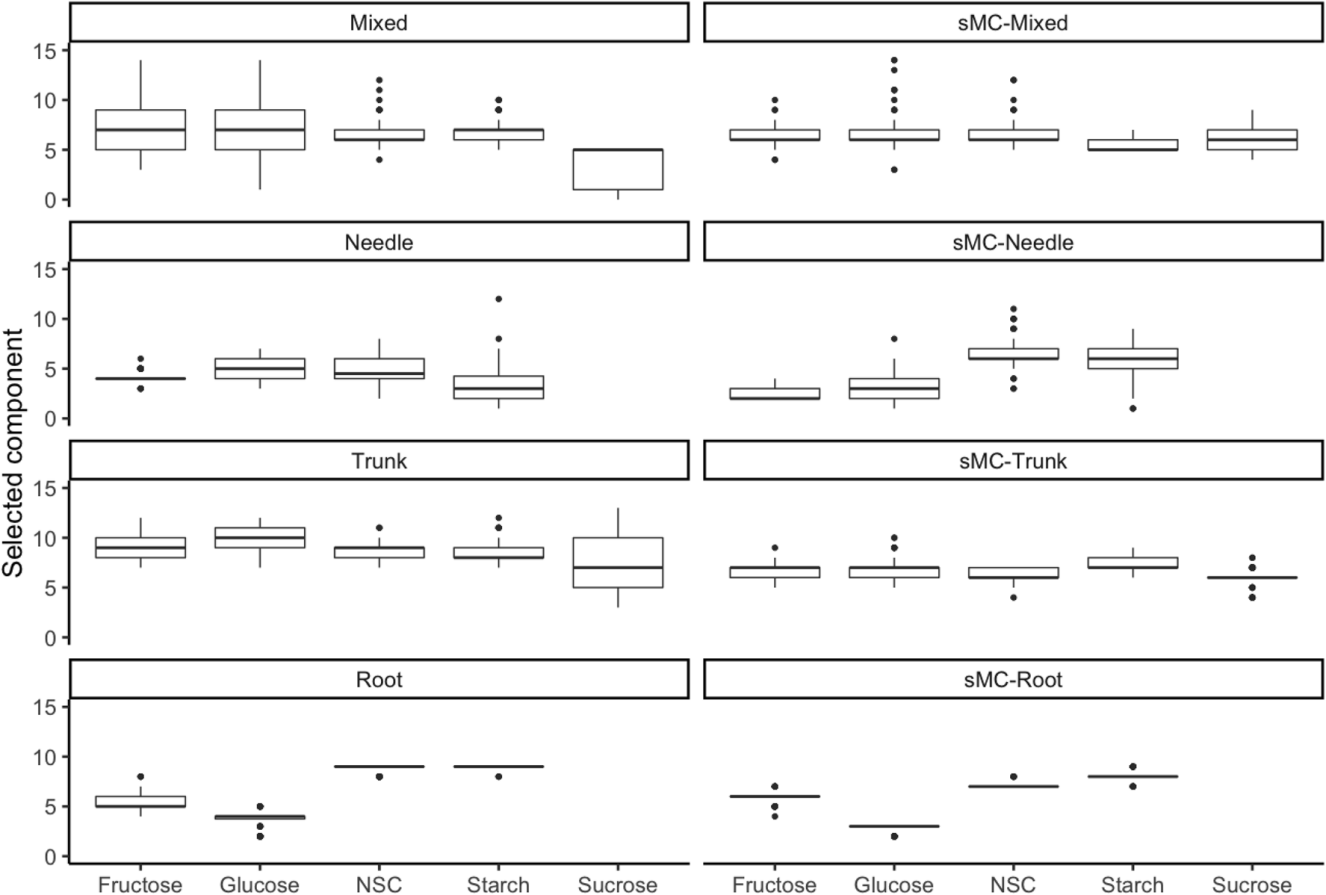
Selected optimal components in each model for NSC, starch, glucose, fructose and sucrose prediction with and without sMC variable selection

**Fig. 6:**
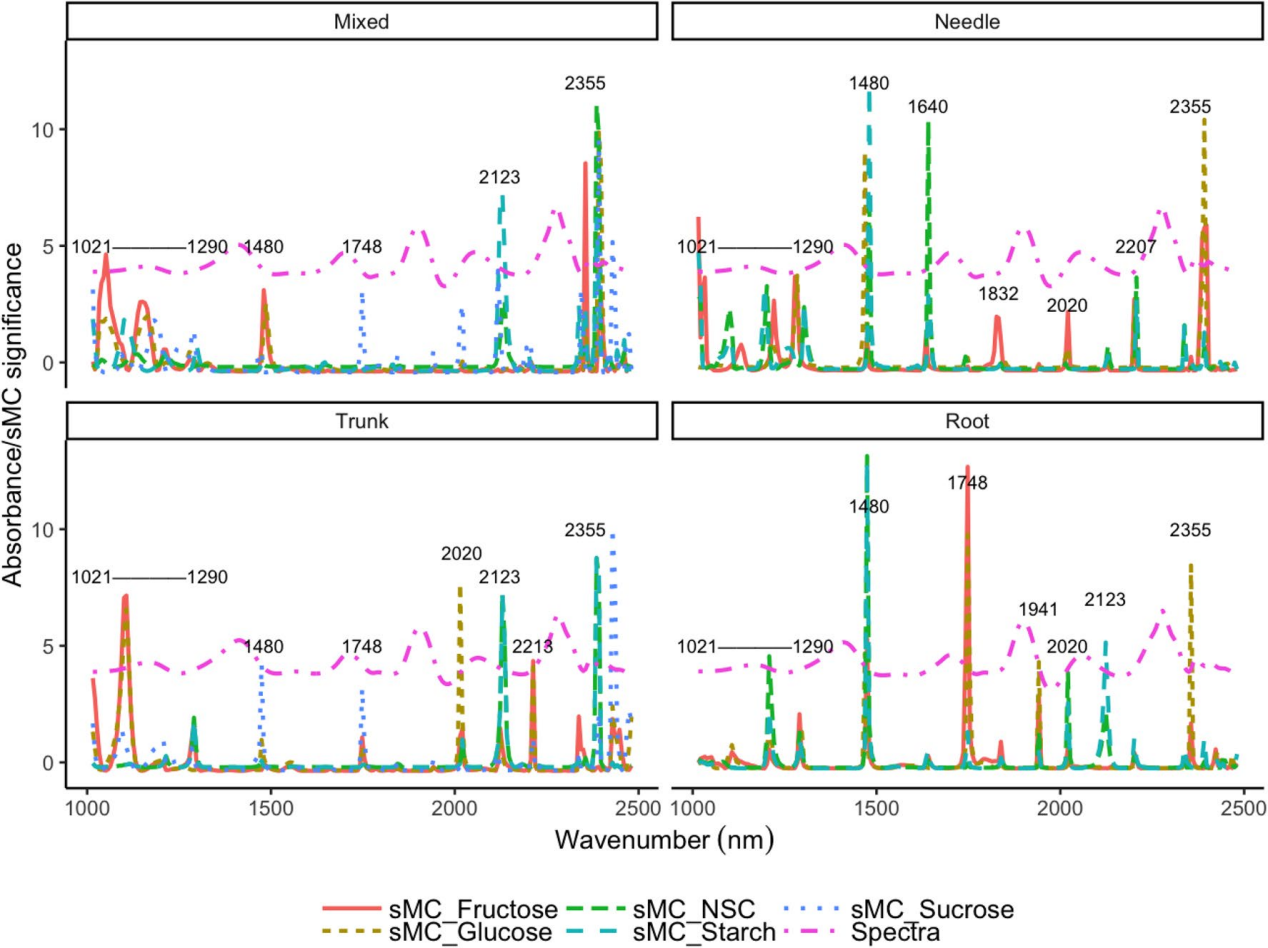
NIR spectra for detection of NSC, starch and free sugars in four slash pine tissue models and the variables selected by the sMC algorithm (upper left: mixed tissues model; upper right: needle model; bottom left: trunk model; bottom right: root model)

The significance multivariate correlation (sMC) algorithm was applied to all PLSR models for variable selection. The number of variables was significantly reduced by the sMC algorithm for all PLSR models, while the optimal number of components remained the same as for the models based on the full spectra (Fig. 5). Similar important variables were selected for the four models: seven most important spectral regions, namely 1021–1290 nm, 1480, 1748, 1941, 2020, 2123 and 2355 nm, were chosen as related to NSC, starch, fructose, glucose and sucrose. NSC and starch shared similar important regions (1480, 1640, 2020, and 2123 nm) while fructose, glucose and sucrose shared the same selected regions (1021–1290, 1748 and 2355 nm) (Fig. 6).

**Fig. 7:**
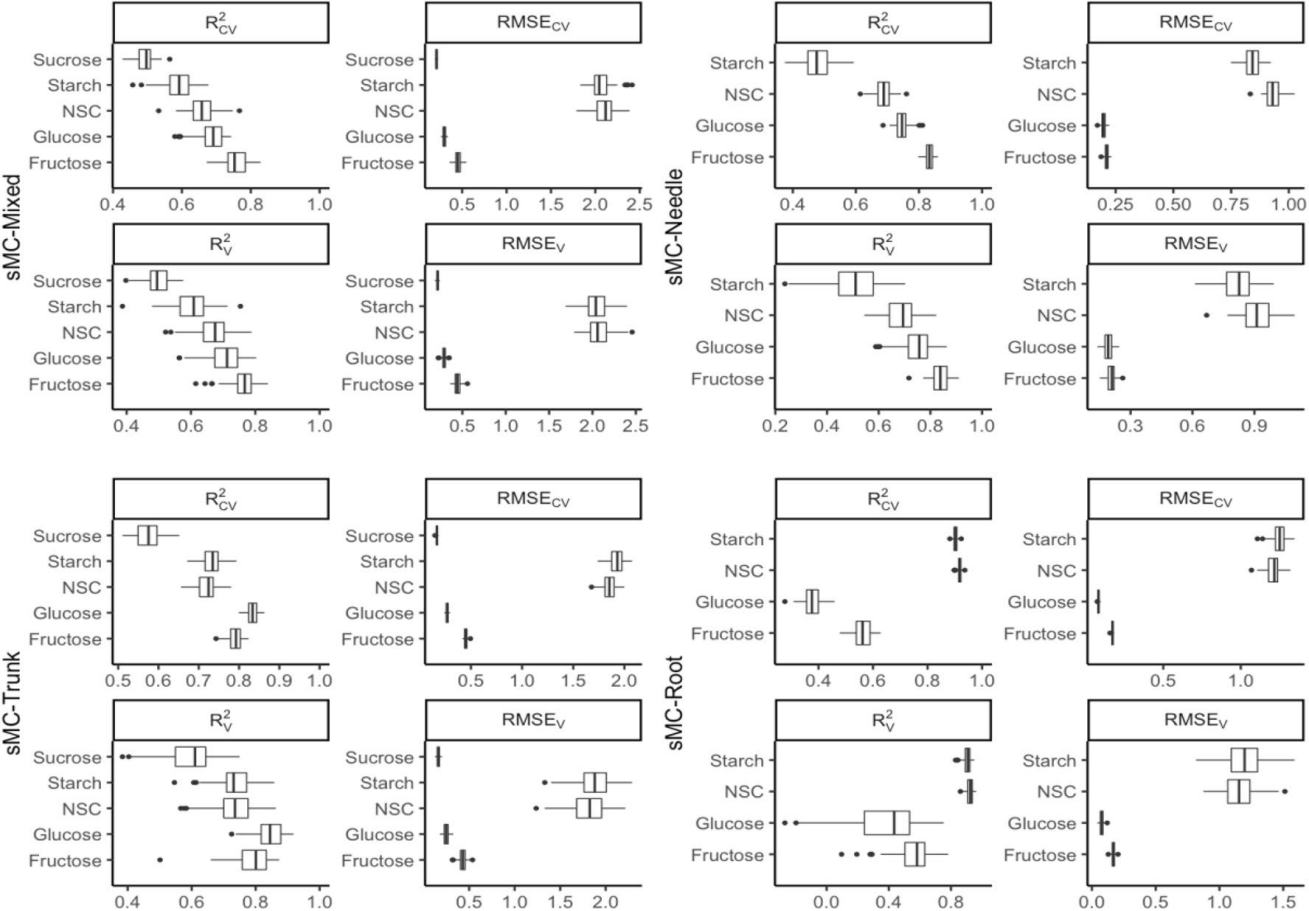
Distribution (95% confidence intervals) of calibration and validation statistics from 100 simulations for models predicting NSC, starch, glucose, fructose and sucrose in plant tissues after sMC variable selection. Each model permutation included 80% of the data for calibration and the remaining 20% for validation. R^2^_CV_: coefficient of determination of cross-validation; R^2^_v_: coefficient of determination of prediction when the model was applied to the validation data set; RMSE_CV_: root-mean-square error of cross-validation; RMSE_v_: root mean-square error of prediction when the model was applied to the validation data set

**Fig. 8:**
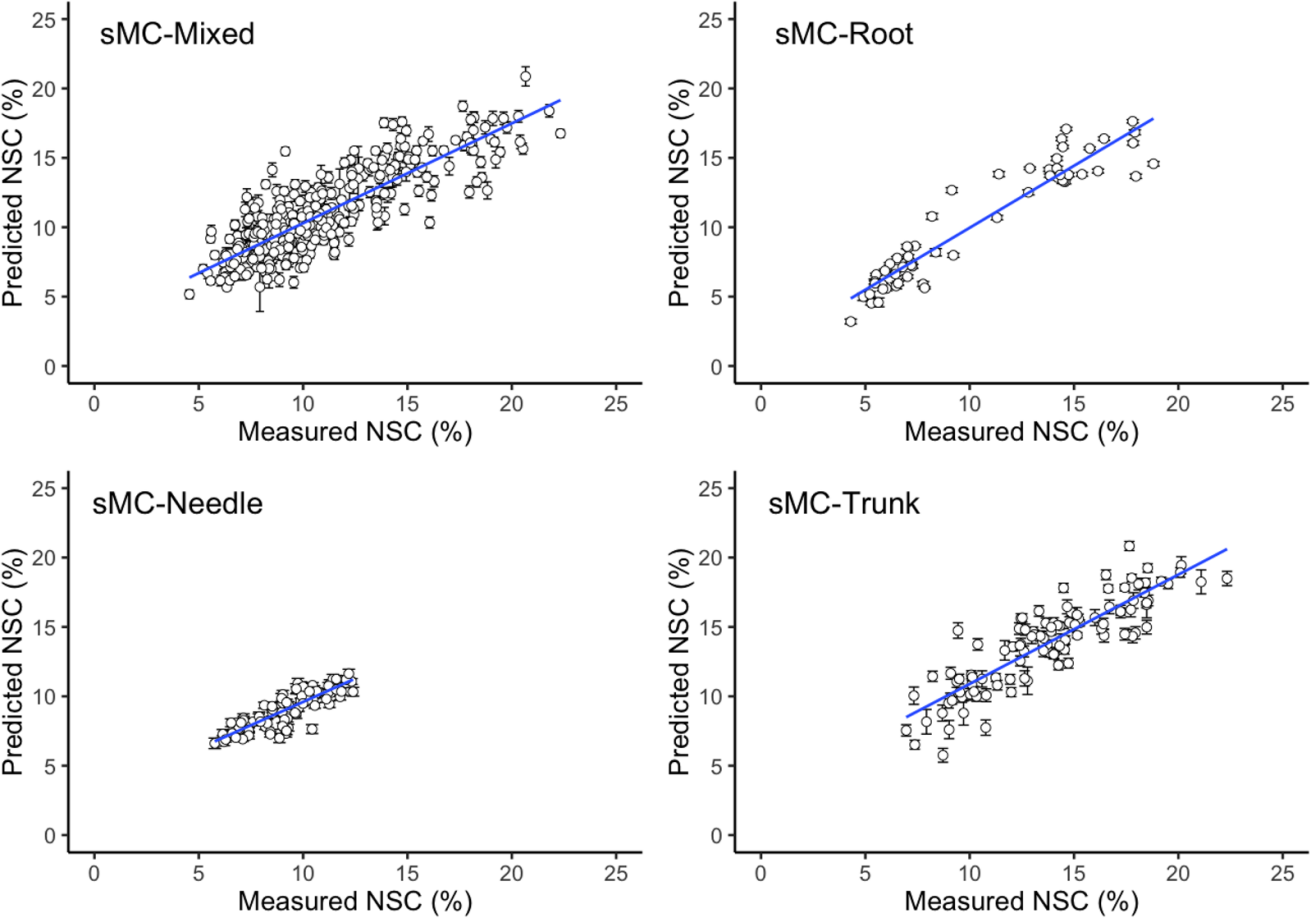
Measured vs. predicted NSC in mixed tissues and single tissue models. Error bars for predicted values represent the standard deviations obtained from 100 simulations using the models

The sMC-PLSR models based on the modified NIR spectra showed better results on both the calibration and validation sets with similar components (Fig. 7). The four different sMC-PLSR models each yielded a satisfactory result to predict NSC in the validation set (Fig. 8).

## Discussion

### NSC allocation

Resin tapping has been applied widely in mature slash pine plantations in China, and we aimed to investigate its influence on NSC allocation in plant tissues. In our study, we found that both tapped and control trees stored more NSC, starch and free sugars in winter than summer: it is likely that trees need more NSC to support growth and metabolism during the growing season and will store more NSC in winter as a resource for future use [41]. The concentrations of NSC, starch and free sugars in trunk phloem and root were reduced after resin tapping in summer, but the concentrations in root were lower in winter in tapped compared to control trees. Tapping was conducted every two days during the summer, and this might have caused increased biosynthesis of resin in existing and newly-induced vertical resin ducts as a defense against external attack. Therefore, more NSC might be needed near the tapping area to support resin production and the formation of traumatic resin ducts [10]. Furthermore, the NSC and starch concentrations in branches and needles of tapping trees were slightly higher than in control trees during the growing season, while older branches and needles contained higher NSC and starch concentrations than newly ones. During the winter, when tapping was stopped, NSC and starch showed similar concentrations in both tapped and control trees. Continuous resin tapping might be a trigger for trees to produce methyl jasmonate, which acts as a sensory signal and stimulates the plant to produce protective compounds. Such metabolism would use NSC from needle photosynthesis and might also stimulate the roots to absorb more mineral nutrients [41, 42]. It was reported that white spruce (*Picea glauca*) trees increased the synthesis of jasmonic acid in roots and leaves to promote increased production of soluble carbohydrates for defense against spruce budworm [43]. However, pine trees, like all plants, usually store NSCs in the trunk and root for metabolism [1]. Trees might first reallocate the stored NSCs near the tapping area to form new defensive structures and synthesize defensive secondary metabolites if the stored NSC is sufficient supply. Any excess newly created NSCs would be stored, which might explain why the NSC concentrations in branches and needles were higher in tapped trees than in control trees. Körner [44] called this response phenomenon sink limitation, meaning that when the demand for carbohydrates is lower than the supply from gross photosynthetic activity, the NSC concentration will increase. Our results differ from other studies which reported that trees use stored NSCs to fuel growth and respiration only when the supply of photoassimilates is insufficient [45–47].

In the tree trunk phloem, resin tapping caused the tapped side to need more NSC resources to produce resin for resistance; more NSCs were transformed into free sugars for reproduction. Therefore, the tapped side might have more free sugars than the non-tapped side; during the dormant season, the free sugars would return to lower levels, and more NSC would be stored in the trunk for future use. However, tapping might reduce the capacity for NSC storage (Fig. 3). In fact, similar studies also observed that *Pinus* species may indicate a more localized traumatic resin canal response after NSC is rapidly attenuated with distance from the attack position [48]. The effects of a localized sink for carbohydrate in bark and wood have been observed in tapped rubber trees [49].

### Predictive models based on NIR signatures

Different data sets were divided into four groups for NIR model calibration, based on the plant tissue type, namely root, trunk phloem, needle and a mixed tissues model. Different ages of needles or branches (newly and one-year-old) were combined together like needle and trunk phloem, respectively. We found we could accurately estimate total NSC and starch distribution in plant tissues using NIR spectroscopic models after variable selection; similar results were reported by Ramirez, Posada, Handa, Hoch, Vohland, Messier and Reu [26]. Rosado, Takarada, Araújo, Souza, Hein, Rosado and Gonçalves [22] also investigated the physiological responses of plants to different environmental stresses, but in *Toona ciliata* M. Roemer var. *australis* wood grown in southern Brazil. They reported promising results for estimating total sugars (R^2^ = 0.88, RMSE = 2.76%), total NSC (R^2^ = 0.90, RMSE = 2.58%), sucrose (R^2^ = 0.82, RMSE = 0.06%) and starch (R^2^ = 0.80, RMSE = 1.03%). Our NIR-based model showed a promising and reliable result for predicting starch in the root (R^2^_CV_ = 0.91 and RMSE_CV_ = 1.49%, Fig. 8), which was similar to the statistics reported for *Rumex obtusifolius* roots (R^2^_CV_ = 0.98 and RMSE = 1.85%) [24]. Free sugars, specifically sucrose in all plant tissues tested and glucose and fructose in root did not yield robust prediction results, which might be because the very small amount of the sugars reduced the variability in these tissues. Low concentrations and variability influenced the accuracy in a previous report of NIR calibration [50]. Conversely, our results for predicting glucose and fructose in the trunk and needle models were similar to the results reported in sorghum stalks, which yielded R^2^ of 0.81 for estimating glucose and sucrose [51].

The accuracy of model predictions for NSC, starch and free sugars varied between plant tissues: for example, we found relatively poor statistics associated with calibrations/validations for sucrose and starch in the trunk and needle models, respectively. Sample types might have a major influence on the model calibration; the analysis of NSC, starch and free sugars might be influenced by plant tissues because of the presence of various primary and secondary metabolites [31]. For example, primary metabolites might lead to biased measurements in NIR spectral collection and secondary metabolites might obscure the absorbance of carbohydrates [26, 52]. These findings suggest that specific chemical compounds and plant tissue types should be considered in future studies when using NIR to predict NSC, starch and free sugars.

The sMC-PLSR models efficiently identified the wavelengths with significant coefficient regressions and enabled us to select a small set of variables to yield a promising and robust predictive model based on NIR data. Our results support those reported by Li and Altaner [53], who successfully used the sMC variable selection method to improve the accuracy of a NIR calibration model to predict concentrations in extracts of the heartwood of *Eucalyptus bosistoana* trees. Other studies also showed that significance multivariate correlation (sMC) is a useful algorithm to remove confounding effects in NIR calibrations [35].

We used a robust statistical methodology for sample selection, which was first used by Couture, Singh, Rubert-Nason, Serbin, Lindroth and Townsend [31] to predict plant secondary metabolites using reflectance spectroscopy. We conducted 100 randomized simulations for calibrating the models to provide an estimate of the model uncertainty and overall stability (Figs. 3, 6, 7). Other studies used either random sampling [54] or the Kennard-Stone sampling algorithm [55]; such methods that sample only once for model calibration may cause instability in models for prediction.

Similar important variables which were related to the NSC, starch, and free sugars were selected in all the models, namely spectral regions close to 1021–1290 nm, 1480, 1640, 1748, 1941, 2020, 2123 and 2355 nm. These regions are mostly associated with O–H and C–H stretching vibrations, as reported by Ramirez, Posada, Handa, Hoch, Vohland, Messier and Reu [26]. For instance, numerous key signals have been reported in the range 1021–1290 nm that are related to the 1st overtones of C–H combination bands and 1st and 2nd overtones of O–H and N–H stretching vibrations, while peaks close to 1480 nm, which are mostly related to the 1st overtones of O–H stretching vibration, are associated with NSC, starch and all free sugars (Fig. 6) [56]. Regions close to 2123, 2355 and 1748 nm were reported as associated with starch and sugar [24].

## Conclusions

In conclusion, in our study, we found that resin tapping induced the production of NSC in plant tissues. However, the responses from each plant tissue were different; the position of resin tapping mainly influenced the dynamic responses of NSC. The NSC allocation and dynamic were strongest close to the tapping position. The tapping area will cause the NSC transfer to free sugars for reproduction. NIR spectroscopy could potentially be used to estimate the NSC and free sugars in trees. However, such studies should concentrate on models using data from specific plant tissues. The repeated spectral statistical methodology that we used provides an efficient way to deal with variation in calibration data and generate information on the responses of plant NSC by using NIR spectra.

## Data Availability

Not applicable.

## References

[1] Hartmann H, Adams HD, Hammond WM, Hoch G, Landhäusser SM, Wiley E, Zaehle S (2018). Identifying differences in carbohydrate dynamics of seedlings and mature trees to improve carbon allocation in models for trees and forests. Environ Exp Bot.

[2] Gessler A, Grossiord C (2019). Coordinating supply and demand: plant carbon allocation strategy ensuring survival in the long run. New Phytol.

[3] Bu WS, Chen FS, Wang FC, Fang XM, Mao R, Wang H (2019). The speciesspecific responses of nutrient resorption and carbohydrate accumulation in leaves and roots to nitrogen addition in a subtropical mixed plantation. Can J For Res.

[4] Kozlowski TT, Kramer PJ, Pallardy SG (2012). The physiological ecology of woody plants.

[5] Deslauriers A, Garcia L, Charrier G, Buttò V, Pichette A, Paré M (2021). Cold acclimation and deacclimation in wild blueberry: direct and indirect influence of environmental factors and nonstructural carbohydrates. Agric For Meteorol..

[6] Sørensen ST, Campbell ML, Duke E, ManleyHarris M (2018). A standard, analytical protocol for the quantitation of nonstructural carbohydrates in seagrasses that permits interlaboratory comparison. Aquat Bot..

[7] Gibon Y, Pyl ET, Sulpice R, Lunn JE, Hoehne M, Guenther M, Stitt M (2009). Adjustment of growth, starch turnover, protein content and central metabolism to a decrease of the carbon supply when Arabidopsis is grown in very short photoperiods. Plant Cell Environ.

[8] Rissanen K, Hölttä T, Bäck J, Rigling A, Wermelinger B, Gessler A (2021). Drought effects on carbon allocation to resin defences and on resin dynamics in old-grown Scots pine. Environ Exp Bot.

[9] Hartmann H, Trumbore S (2016). Understanding the roles of nonstructural carbohydrates in forest trees–from what we can measure to what we want to know. New Phytol.

[10] Lombardero M, Ayres MP, Ruel JJ (2000). Environmental effects on constitutive and inducible resin defences of *Pinus taeda*. Ecol Lett.

[11] LópezVillamor A, Carreño S, LópezGoldar X, SuárezVidal E, Sampedro L, Nordlander G, Björklund N, Zas R (2019). Risk of damage by the pine weevil *Hylobius abietis* in southern Europe: effects of silvicultural and landscape factors. For Ecol Manag.

[12] de Oliveira Junkes CF, Duz JVV, Kerber MR, Wieczorek J, Galvan JL, Fett JP, Fett-Neto AG (2019). Resinosis of young slash pine (*Pinus **elliottii* Engelm.) as a tool for resin stimulant paste development and high yield individual selection. Ind Crops Prod.

[13] Zhang S, Jiang J, Luan Q (2016). Genetic and correlation analysis of oleoresin chemical components in slash pine. Genet Mol Res.

[14] Neis FA, de Costa F, Füller TN, de Lima JC, da Silva Rodrigues-Corrêa KC, Fett JP, Fett-Neto AG (2018). Biomass yield of resin in adult *Pinus **elliottii* Engelm. trees is differentially regulated by environmental factors and biochemical effectors. Ind Crops Prod.

[15] Zeng X, Sun S, Wang Y, Chang Y, Tao X, Hou M, Wang W, Liu X, Zhang L (2021). Does resin tapping affect the tree-ring growth and climate sensitivity of the Chinese pine (*Pinus tabuliformis*) in the Loess Plateau, China?. Dendrochronologia.

[16] Reta Z, Adgo Y, Girum T, Mekonnen N (2020). Assessment of contribution of non-timber forest products in the socio-economic status of peoples in Eastern Ethiopia. Open Access J Biogener Sci Res.

[17] Meinhold K, Darr D (2019). The processing of non-timber forest products through small and medium enterprises—a review of enabling and constraining factors. Forests.

[18] Rijkers T, Ogbazghi W, Wessel M, Bongers F (2006). The effect of tapping for frankincense on sexual reproduction in *Boswellia papyrifera*. J Appl Ecol.

[19] Oliva J, Stenlid J, Martínez-Vilalta J (2014). The effect of fungal pathogens on the water and carbon economy of trees: implications for drought-induced mortality. New Phytol.

[20] Mengistu T, Sterck FJ, Fetene M, Bongers F (2013). Frankincense tapping reduces the carbohydrate storage of Boswellia trees. Tree Physiol.

[21] Türker-Kaya S, Huck CW (2017). A review of mid-infrared and near-infrared imaging: principles, concepts and applications in plant tissue analysis. Molecules.

[22] Rosado LR, Takarada LM, Araújo ACCD, Souza K, Gonalves F (2019). Near infrared spectroscopy: rapid and accurate analytical tool for prediction of nonstructural carbohydrates in wood. Cerne.

[23] Petisco C, GarciaCriado B, Mediavilla S, De Aldana BV, Zabalgogeazcoa I (2006). Nearinfrared reflectance spectroscopy as a fast and nondestructive tool to predict foliar organic constituents of several woody species. Anal Bioanal Chem.

[24] Decruyenaere V, Clément C, Agneessens R, Losseau C, Stilmant D (2012). Development of near-infrared spectroscopy calibrations to quantify starch and soluble sugar content in the roots of *Rumex obtusifolius*. Weed Res.

[25] RuizAquino F, GonzálezPeña MM, ValdezHernández JI, Revilla US, RomeroManzanares A (2015). Chemical characterization and fuel properties of wood and bark of two oaks from Oaxaca, Mexico. Ind Crops Prod.

[26] Ramirez JA, Posada JM, Handa IT, Hoch G, Vohland M, Messier C, Reu B (2015). Near-infrared spectroscopy (NIRS) predicts non-structural carbohydrate concentrations in different tissue types of a broad range of tree species. Methods Ecol Evol.

[27] Aw WC, Ballard JWO (2019). Near-infrared spectroscopy for metabolite quantification and species identification. Ecol Evol.

[28] Sytar O, Bruckova K, Hunkova E, Zivcak M, Konate K, Brestic M (2015). The application of multiplex fluorimetric sensor for the analysis of flavonoids content in the medicinal herbs family Asteraceae, Lamiaceae, Rosaceae. Biol Res.

[29] Sytar O, Zivcak M, Neugart S, Brestic M (2020). Assessment of hyperspectral indicators related to the content of phenolic compounds and multispectral fluorescence records in chicory leaves exposed to various light environments. Plant Physiol Biochem.

[30] RodríguezGarcía A, Martín JA, López R, Mutke S, Pinillos F, Gil L (2015). Influence of climate variables on resin yield and secretory structures in tapped *Pinus pinaster* Ait in central Spain. Agric For Meteorol.

[31] Couture JJ, Singh A, RubertNason KF, Serbin SP, Lindroth RL, Townsend PA (2016). Spectroscopic determination of ecologically relevant plant secondary metabolites. Methods Ecol Evol..

[32] Tümen İ, Reunanen M (2010). A comparative study on turpentine oils of oleoresins of *Pinus sylvestris* L from three districts of Denizli. Rec Nat Prod.

[33] Hoch G, Richter A, Körner C (2003). Non‐structural carbon compounds in temperate forest trees. Plant, Cell &amp; Environment.

[34] Mehmood T, Liland KH, Snipen L, Sæbø S (2012). A review of variable selection methods in partial least squares regression. Chemometr Intell Lab Syst..

[35] Tran TN, Afanador NL, Buydens LM, Blanchet L (2014). Interpretation of variable importance in partial least squares with significance multivariate correlation (sMC). Chemometr Intell Lab Syst..

[36] R Core Team R: a language and environment for statistical computing, Vienna, Austria, 2017.

[37] Mevik B, Wehrens R, Hovde L. partial least squares and principal component regression. R package version 260 2015.

[38] Stevens A, Ramirez–Lopez L. An introduction to the prospectr package. R package version 013 2014.

[39] Wickham H. ggplot2: Elegant Graphics for Data Analysis. SpringerVerlag New York 2009.

[40] Butler D, Cullis B, Gilmour A, Gogel B, Thompson R. ASReml-R Reference Manual Version 4.1. 0.130. In.: VSN International Ltd, https://asreml.kb.vsni.co.uk; 2020.

[41] Schoonmaker A, Hillabrand R, Lieffers V, Chow P, Landhäusser S (2021). Seasonal dynamics of non-structural carbon pools and their relationship to growth in two boreal conifer tree species. Tree Physiol.

[42] Celedon JM, Bohlmann J (2019). Oleoresin defenses in conifers: chemical diversity, terpene synthases and limitations of oleoresin defense under climate change. New Phytol.

[43] Mageroy MH, Parent G, Germanos G, Giguère I, Delvas N, Maaroufi H, Bauc É, Bohlmann J, Mackay JJ (2015). Expression of the β-glucosidase gene Pgβglu-1 underpins natural resistance of white spruce against spruce budworm. Plant J.

[44] Körner C (2003). Carbon limitation in trees. J Ecol.

[45] Carbone MS, Trumbore SE (2007). Contribution of new photosynthetic assimilates to respiration by perennial grasses and shrubs: residence times and allocation patterns. New Phytol.

[46] Kuptz D, Fleischmann F, Matyssek R, Grams TEE (2011). Seasonal patterns of carbon allocation to respiratory pools in 60-yr-old deciduous (*Fagus sylvatica*) and evergreen (*Picea abies*) trees assessed via whole-tree stable carbon isotope labeling. New Phytol.

[47] Richardson AD, Carbone MS, Keenan TF, Czimczik CI, Hollinger DY, Murakami P, Schaberg PG, Xu X (2013). Seasonal dynamics and age of stemwood nonstructural carbohydrates in temperate forest trees. New Phytol.

[48] Krokene P, Nagy NE. Anatomical aspects of resin based defences in pine. Pine Resin Biol Chem Appl. 2012; 67–86.

[49] Ansari AH, Jakarni FM, Muniandy R, Hassim S, Elahi Z (2020). Natural rubber as a renewable and sustainable bio-modifier for pavement applications: a review. J Clean Prod.

[50] Cortés V, Blasco J, Aleixos N, Cubero S, Talens P (2019). Monitoring strategies for quality control of agricultural products using visible and near-infrared spectroscopy: a review. Trends Food Sci Technol.

[51] Chen SF, Danao MGC, Singh V, Brown PJ (2014). Determining sucrose and glucose levels in dual-purpose sorghum stalks by Fourier transform near infrared (FT-NIR) spectroscopy. J Sci Food Agric.

[52] Workman J, Weyer L (2012). Practical guide and spectral atlas for interpretive nearinfrared spectroscopy.

[53] Li Y, Altaner C (2018). Predicting extractives content of *Eucalyptus **bosistoana* F Muell Heartwood from stem cores by near infrared spectroscopy. Spectrochim Acta A Mol Biomol Spectrosc..

[54] Quentin AG, Rodemann T, Doutreleau MF, Moreau M, Davies NW, Millard P (2017). Application of nearinfrared spectroscopy for estimation of nonstructural carbohydrates in foliar samples of *Eucalyptus globulus* Labilladière. Tree Physiol.

[55] Li Z, Li C, Gao Y, Ma W, Zheng Y, Niu Y, Guan Y, Hu J (2018). Identification of oil, sugar and crude fiber during tobacco (*Nicotiana tabacum* L) seed development based on near infrared spectroscopy. Biomass Bioenergy.

[56] Schwanninger M, Rodrigues JC, Fackler K (2011). A review of band assignments in near infrared spectra of wood and wood components. J Near Infrared Spectrosc.

